# Elucidating brain metabolic changes during short-term fasting: a comprehensive atlas

**DOI:** 10.3389/fnins.2023.1334461

**Published:** 2024-01-03

**Authors:** Yang Ni, Murad Al-Nusaif, Yiying Hu, Tianbai Li

**Affiliations:** Liaoning Provincial Key Laboratory for Research on the Pathogenic Mechanisms of Neurological Diseases, The First Affiliated Hospital of Dalian Medical University, Dalian, Liaoning, China

**Keywords:** metabolome, short-term fasting, brain region, brain health, neurodegenerative disorders

## Introduction

**Figure 1:**
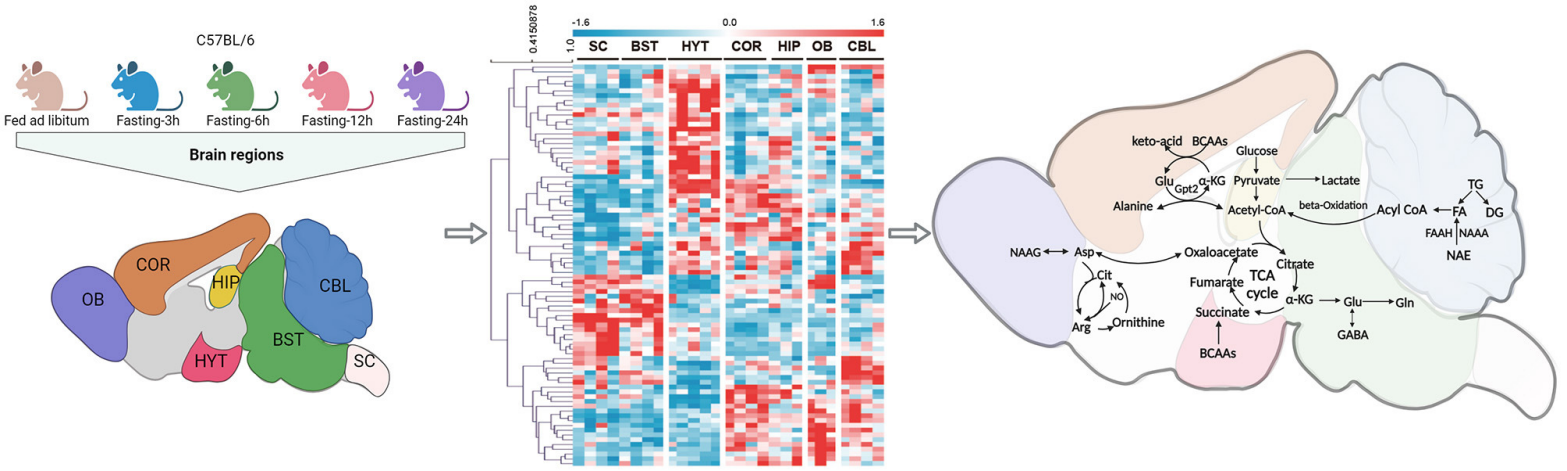
Metabolomic analysis of seven brain regions in C57BL/6 mice under fasting conditions (3 h, 6 h, 12 h, and 24 h) and *ad libitum* feeding. A heat map was presented to illustrate the differential expression of metabolites in these seven brain regions (Shao et al., 2023). The analysis revealed that short-term fasting induced a global lipid and amino acid metabolism remodeling, mainly targeting FAs, BCAAs, Glu, GABA, and the citrulline–NO cycle. OB, olfactory bulb; COR, frontal cortex; HYT, hypothalamus; HIP, hippocampus; CBL, cerebellum; BST, brainstem; SC, spinal cord; FAs, fatty acids; BCAAs, branched-chain amino acids; Glu, glutamic acid; GABA, gamma-aminobutyric acid.

The recent study, published in Signal Transduction and Targeted Therapy by Le's team (Shao et al., 2023), provides a comprehensive and detailed metabolome atlas of the mouse brain based on the global metabolic signature dynamics across multiple brain regions in response to short-term fasting (STF) (Shao et al., 2023). This research revealed significant region-specific metabolic remodeling involving lipids, saccharides, and amino acid neurotransmitters under food deprivation (Figure 1) (Shao et al., 2023). These discoveries furnish a crucial molecular foundation and novel insights into the mechanisms through which STF improves brain health.

In recent years, STF has gained prominence as a potent non-pharmacological intervention for weight reduction, attenuating the onset of metabolic and age-related disorders, reducing cancer incidence, and extending lifespans (Varady et al., 2022). As a short-period calorie restriction (CR) form, STF is generally considered safe and is associated with minimal adverse effects on gastrointestinal, neurological and hormonal functions (Varady et al., 2022). Moreover, preclinical and clinical investigations have demonstrated the advantageous effects of STF in conditions such as epilepsy, ischemic stroke, neurodevelopmental and neuropsychiatric disorders, as well as neurodegenerative disorders, including Alzheimer's disease and Parkinson's disease (Gudden et al., 2021). However, the precise mechanisms underlying how STF improves brain health remain partially elucidated. Metabolites, identifiable markers susceptible to alteration during CR, assume a central role in maintaining cerebral homeostasis, which is essential for optimal brain function (Shao and Le, 2019). Therefore, investigating the metabolic reprogramming triggered by STF in the brain holds the potential to provide valuable perspectives into devising effective therapeutic strategies for age-related neurological disorders and neurodegenerative diseases (Le, 2021). The influence of STF on the spatiotemporal brain metabolome remains insufficiently explored, as prior investigations have predominantly focused on a restricted set of metabolites or confined their examination to specific anatomical brain regions (Gudden et al., 2021).

In this study, Le's group conducted comprehensive metabolomics and lipidomics analyses on seven brain regions of mice subjected to different periods of fasting (3 h, 6 h, 12 h, and 24 h). They employed gas chromatography–mass spectrometry (GC-MS) and liquid chromatography-mass spectrometry (LC-MS) techniques, encompassing the profiling of 797 structurally identified metabolites (Figure 1) (Shao et al., 2023). The results unveiled distinctive metabolic signatures within different functional brain regions, highlighting the emergence of a unique repertoire of metabolites in response to STF. Notably, significant variations in metabolites and lipids were discerned in the hippocampus (HIP), olfactory bulb (OB), and spinal cord (SC) regions. The principal metabolic adjustments triggered by STF primarily encompass increased triacylglycerol degradation and heightened fatty acid (FA) production across most brain regions (Shao et al., 2023). FAs assume a pivotal role in upholding cell membrane integrity and function. Typically, they are stored as triacylglycerols within intracellular lipid droplets (LDs) (Bogie et al., 2020). Due to the limited capacity for FA catabolism in neurons, LDs act as vehicles for FA transport to astrocytic mitochondria during fasting. The breakdown of LDs releases FAs, which are subsequently harnessed as an energy source through oxidative phosphorylation in astrocytes, thereby serving an energetic function and mitigating lipotoxicity (Bogie et al., 2020). Recent research further substantiates these findings by observing significant alterations in genes associated with the lipolysis process in the hypothalamus of fasting mice (Oh et al., 2023). This observation provides additional support for the concept of heightened lipolysis occurring within the brain during STF. Furthermore, Le's group discovered that STF triggered a pervasive increase in endogenous lipid N-acylethanolamine levels (Shao et al., 2023). This elevation assumes significance due to the pivotal role played by N-acylethanolamines in cell signaling and cytoprotection. Notably, these compounds exhibit multifaceted functions, including the modulation of mood and cognitive function, facilitation of motor function recovery following cerebral ischemia, provision of neuroprotection against oxidative stress, and mitigation of hippocampus-dependent memory impairment (Shao and Le, 2019).

In addition to lipids, this study has identified noteworthy alterations in amino acid profiles within the brain that may have a significant impact on energy metabolism, neurotransmitter signaling, as well as anti-inflammatory and antioxidant responses to STF (Shao et al., 2023). Specifically, STF was found to elevate the concentrations of branched-chain amino acids (BCAAs) across seven brain regions, as well as glutamic acid (Glu) level in the spinal cord and cerebellum and gamma-aminobutyric acid (GABA) level in the hippocampus and hypothalamus (Shao et al., 2023). These amino acids serve as substrates for energy metabolism by supplementing intermediate metabolites for the tricarboxylic acid (TCA) cycle. GABA is synthesized through Glu decarboxylase, and both Glu and GABA play critical roles in neurotransmission within neurons and astrocytes through the Glu/GABA-glutamine cycle (Sperringer et al., 2017). Furthermore, an analysis of RNA-sequencing data revealed a significant increase in Gpt2, a gene responsible for encoding alanine aminotransferase, across different brain regions during STF (Shao et al., 2023). Alanine aminotransferase facilitates the conversion of pyruvate to alanine, while consuming Glu, thus mitigating damage to the central nervous system from Glu excitotoxicity (Sperringer et al., 2017). Additionally, other significantly altered amino acids during STF, N-acetyl-L-aspartic acid and N-acetyl-aspartyl glutamic acid (NAAG), which have been found to modulate glutamate release by activating presynaptic metabotropic glutamate receptor 3 (mGluR3) (Morland and Nordengen, 2022). Moreover, elevations in citrulline and ornithine levels were noted during fasting, with transcriptome data hinting at the potential activation of the citrulline–nitric oxide (NO) cycle during STF. The role of NO in reducing core body temperature is particularly advantageous in the context of STF (Parkhitko et al., 2019). Additionally, STF appears to lead to decreased methionine levels, a phenomenon associated with documented benefits, including extended lifespan, reduced inflammation, and inhibition of tumor growth (Parkhitko et al., 2019).

Overall, this study's findings have illuminated novel insights into the molecular foundations and mechanisms that underlie STF, thereby providing a valuable foundation for future research regarding the metabolic modifications within the brain during fasting and their potential implications for brain health. Considering the intricate nature of the brain's structure and the intricate network interconnections between distinct brain regions, forthcoming research should consider expanding its focus to encompass more specific brain regions. This study exclusively utilized female mice in early adulthood, limiting the exploration of potential variations in metabolic responses to STF associated with age, hormonal fluctuations, and gender. Employing mice with diverse age groups, genders, and hormonal profiles could furnish supplementary data for elucidating the mechanisms through which STF enhances overall health. Nevertheless, the translation of these metabolic adaptations to human subjects remains uncertain.

## Author contributions

YN: Writing—original draft, Writing—review & editing. M-AN: Writing—original draft, Writing—review & editing. YH: Writing—original draft, Writing—review & editing. TL: Writing – review & editing.
